# Neonatal Hyperbilirubinemia in infants with *G6PD c.563C > TVariant*

**DOI:** 10.1186/1471-2431-12-126

**Published:** 2012-08-20

**Authors:** Bushra Moiz, Amna Nasir, Sarosh Ahmed Khan, Salima Amin Kherani, Maqbool Qadir

**Affiliations:** 1 Department of Pathology and Microbiology, The Aga Khan University Hospital, Karachi, Pakistan; 2 Medical Student, The Aga Khan Medical College, Karachi, Pakistan; 3 Department of Pediatrics and Child Health, The Aga Khan University, Karachi, Pakistan

## Abstract

**Background:**

There is a strong correlation between glucose-6-phosphate dehydrogenase (G6PD) deficiency and neonatal hyperbilirubinemia with a rare but potential threat of devastating acute bilirubin encephalopathy. G6PD deficiency was observed in 4–14% of hospitalized icteric neonates in Pakistan. *G6PD c.563C > T* is the most frequently reported variant in this population. The present study was aimed at evaluating the time to onset of hyperbilirubinemia and the postnatal bilirubin trajectory in infants having *G6PD c.563C > T.*

**Methods:**

This was a case–control study conducted at The Aga Khan University, Pakistan during the year 2008. We studied 216 icteric male neonates who were re-admitted for phototherapy during the study period. No selection was exercised. Medical records showed that 32 were G6PD deficient while 184 were G6PD normal. Each infant was studied for birth weight, gestational age, age at the time of presentation, presence of cephalhematoma, sepsis and neurological signs, peak bilirubin level, age at peak bilirubin level, days of hospitalization, whether phototherapy or exchange blood transfusion was initiated, and the outcome. During hospital stay, each baby was tested for complete blood count, reticulocyte count, ABO and Rh blood type, direct antiglobulin test and quantitative G6PD estimation [by kinetic determination of G6PDH]. *G6PDgenotype* was analyzed in 32 deficient infants through PCR-RFLP analysis and gene sequencing.

**Results:**

*G6PD variants c.563C > T* and *c.131 C > G* were observed in 21 (65%) and three (9%) of the 32 G6PD deficient infants, respectively. DNA of eight (25%) newborns remained uncharacterized. In contrast to G6PD normal neonates, infants with *c.563C > T* variant had significantly lower enzyme activity (mean ± 1SD; 0.3 ± 0.2 U/gHb vs. 14.0 ± 4.5 U/gHb, *p* < 0.001) experienced higher peak levels of total serum bilirubin (mean ± 1SD; 16.8 ± 5.4 mg/dl vs. 13.8 ± 4.6 mg/dl, *p =* 0.008) which peaked earlier after birth (mean ± 1SD 2.9 ± 1.6 vs. 4.3 ± 2.3 days, *p =* 0.007). No statistically significant difference was observed in mean weight, age at presentation, hemoglobin, reticulocyte count, TSH level, hospital stay or in the frequency of initiation of phototherapy or blood exchange between the two groups.

**Conclusions:**

We concluded that infants with *G6PD c.563C > T* variant developed jaundice earlier than infants with normal G6PD enzyme levels. Compared to G6PD normal infants, *G6PD c.563C > T* carrying infants had significantly low G6PD activity.

## Background

G6PD deficiency is the most common red cell enzymopathy estimated to affect 400 million people worldwide [1]. A recent systematic review showed a global prevalence of 4.9% for G6PD deficiency [2]. There is significant association of G6PD deficiency with neonatal hyperbilirubinemia in the immediate perinatal period [3]. Though rare, significant hyperbilirubinemia poses a potential threat for permanent neurological deficit or kernicterus. Studies indicate that insufficient hepatic metabolism of unconjugated bilirubin [4] rather than increased hemolysis [5] is the major contributor to neonatal hyperbilirubinemia. In addition, the UGT1A1 mutation of promoter or coding region in *G6PD* contributes to a Gilbert like condition [6,7] in G6PD deficient infants. To date 400 biochemical G6PD variants have been identified corresponding to 186 G6PD mutations [8], with most being single point mutations. Recent advances in technology have permitted accurate molecular characterization in many regions of the globe. However, few reports (primarily from Chinese populations) have investigated the relationship between *G6PD* variants and the severity of neonatal hyperbilirubinemia [9-11], while others focused only on identification of *G6PD* variants in icteric infants [12-14].

Two large national Pakistani studies (n = 1624 and 6454 patients respectively) reported that 26% [15] and 30% [16] of all hospital admissions were required for evaluation of neonatal jaundice. Low birth weight, ABO or Rh incompatibility and sepsis were recognized as important contributors for jaundice [15] while G6PD deficiency was observed in 8% of jaundiced infants [16]. With two thirds of infants in Pakistan being born outside hospitals, the true magnitude of neonatal hyperbilirubinemia is expected to be much higher than observed in these studies. Reported incidence of G6PD deficiency in Pakistani males ranges from 2 to 4% [17-26] with a higher incidence of 8% in Pathans. *G6PD c.563C > T* is the most frequent variant [21,27]. National literature review indicated a higher prevalence [4 to14%] of G6PD deficiency in jaundiced neonates [16,28-33]. These reports also showed that the infants developed jaundice within their first five days of life and a substantial number of them required phototherapy and exchange blood transfusions [34]. Unfortunately up to 22% suffered from acute bilirubin encephalopathy and their mortality was as high as 4% [16,30]. Despite extensive study of G6PD deficiency in Pakistani neonates, there has been no national interest in molecular characterization of *G6PD gene*. For example, we don’t know whether infants with various *G6PD variants* behave differently.

*G6PD c.563C > T* is the most frequent variant in Pakistan [21,27]. Because it is associated with very low enzyme activity [1], we hypothesized that the neonates inheriting this variant would exhibit severe hyperbilirubinemia requiring more aggressive management compared to icteric infants having normal G6PD activity. The present study was aimed at evaluating the time to onset of hyperbilirubinemia and the postnatal bilirubin trajectory in infants having *G6PD* c.563C > T.

## Methods

### Protocol for management of hyperbilirubinemia

Situated in Southern Pakistan, The Aga Khan University is an academic tertiary care hospital with advanced neonatal care facilities. Over 600 neonates are admitted annually to the neonatal intensive care unit (NICU) and treated for various disorders including hyperbilirubinemia. Our institution follows the guidelines laid by American Academy of Pediatrics for management of neonatal hyperbilirubinemia [29]. Hyperbilirubinemia was defined as a serum total bilirubin [STB] of >15 mg/dl in the first week of life and infant’s age was measured in hours and approximated to days. Infants were assessed for jaundice every 8–12 h by our medical and nursing staff. Indications for STB estimation included: onset of jaundice in first 24 h, excessive jaundice for age and deepening or unexplained jaundice [29]. Blood was drawn at 48 h in all infants for mandatory bilirubin determination. For designation of risk, hour-specific STB was plotted on Bhutani’s nomogram [29]. Subsequent blood draws were made daily between 6–8 am to avoid systematic bias. More frequent STB estimations were done for infants in moderate and higher risk zones. Common causes of pathologic hyperbilirubinemia considered in each neonate included ABO or Rh incompatibly, sepsis, hematomas, prematurity, hypothyroidism [35]. ABO or Rh incompatibility was identified by a positive direct antiglobulin test in an infant born to a blood group O or Rh negative mother. Sepsis was defined as systemic inflammatory response syndrome associated with suspected or proven infection. Criteria included: a core temperature of > 38.5°C or < 36°C, a heart rate of > 180 or < 100/min, a respiratory rate of > 50/min, systolic blood pressure of < 65 mmHg and white cell count of < 5 or >34 × 10^9^/l [36]. Elevated C-reactive protein of > 10 mg/L was considered as a biochemical marker for infection [37]. Additionally thrombocytopenia < 80 × 10^9^/l was observed as a marker of severe sepsis [36]. Published normograms correlating age [in hours] and STB were utilized for evaluating the need of phototherapy or exchange transfusion [29].G6PD enzyme test was performed for all babies who were re-admitted for phototherapy. TSH assessment was done routinely as an integral component of the neonatal screening program irrespective of icterus. Infants were discharged upon demonstrating resolution of clinical jaundice with declining STB. Follow-up visits were scheduled for all infants.

### Subjects studied

Computerized hospital data indicated 455 neonates with hyperbilirubinemia during January 1 to December 31, 2008. There were 270 icteric neonates who were re-admitted in NICU for phototherapy. After excluding female infants [n = 52], cases with missing data and incomplete information [n = 2], medical records were reviewed for 216 icteric babies. All 216 babies were tested for G6PD enzyme activity.

Clinical Details: The following details were extracted from the medical record of all neonates: birth weight, gestational age at birth, age at the time of presentation of jaundice, presence of cephalhematoma, sepsis and neurological signs, peak bilirubin level, age at peak bilirubin level, days of hospitalization, whether phototherapy was initiated, and outcome.

Laboratory parameters: During hospital stay**,** each baby was tested for complete blood count [Coulter® Gen-S; Coulter Electronics, Hialeah FL, USA], reticulocyte count[Coulter® Gen-S; Coulter Electronics, Hialeah FL, USA], ABO and Rh blood group [Classic DiaMed- ID, Cressier, Switzerland], direct antiglobulin test [Classic DiaMed- ID, Cressier, Switzerland], total direct and indirect bilirubin levels [Synchron, Beckman, Coulter®, USA] and serum TSH [E170, Roche®, Germany]. G6PD quantitative enzyme assay [Trinity®, Biotech kit No 345, Wicklow, Ireland] was normalized using the patient’s hemoglobin according to manufacturer’s instructions. The test involved kinetic determination of G6PDH in blood at 340 nm. The neonatal reference interval for G6PD has been previously established in our laboratory, and ranges from 7 to19 U/gHb; G6PD deficiency was diagnosed when an enzyme level was < 7 U/gHb.

### Molecular analysis

The blood samples sent for G6PD enzyme assay were saved for molecular analysis if quantitative enzyme deficiency was observed. DNA was extracted from white cells using Quigen® kit and a three steps strategy was used to characterize *G6PD* at molecular level. All samples were scanned for *c.563C > T* mutation and *c.1311C > T* polymorphism by restriction fragment length polymorphism (PCR-RFLP). Failure to detect *c.563C > T* prompted for a more detailed PCR analysis for nine common mutations prevalent in South Asia: c.*95A > G, c.1311C > T, c.392 G > T, c.871 G > A, c.1003 A > G, c.1024 C > T, c.1376 G > C, c.1376 G > T and c.1388 G > A*[21,38-41]*.* When a sample remained uncharacterized after these first two steps, more comprehensive genetic analysis with amplification of 9–12 exons [42] was performed followed by sequence analysis (Macrogen®, Soule, Korea). G6PD genotype of non-deficient infants was not analyzed. *G6PD c.563C > T* is associated with very low levels of enzyme activity which is usually < 1 U/gHb [43]. Therefore it was assumed that this variant would not be present in G6PD normal infants.

### Statistical analysis

All the data was entered into SPSS version 16 [SPSS Inc., Chicago, IL, USA] for analysis. Continuous as well as discrete data were compared for G6PD normal, deficient and *G6PD c.563C > T* variant utilizing Student’s *T test* if the distribution was normal. The threshold of significance was considered as a *p* < 0.05.

### Ethical concerns

The study was approved by institutional ethical review committee of The Aga Khan University Hospital [ERC approval No.# Pat 506/ERC 06]. Informed consent was taken from the parents and the data was entered after recoding to maintain anonymity. Parents were informed of the possibility of mutation remaining unidentified.

## Results

### Demographics

We studied 216 icteric male infants who were re-admitted for phototherapy. G6PD deficiency was observed in 32 babies [15%] with a low G6PD enzyme activity of 0.6 ± 0.9 U/gHb. There were 184 infants who had normal G6PD levels with a mean ± 1SD of 14.0 ± 4.5 U/gHb. The variables of 184 G6PD normal and 32 G6PD deficient male infants are summarized in Table 1. G6PD deficient infants developed icterus earlier than G6PD normal infants (Mean ± 1SD; 1.6 ± 1.3 vs. 2.2 ± 2.2 days, p = 0.026).

**Table 1:** Clinical and laboratory parameters of jaundiced males infants with (n = 32) and without G6PD deficiency (n = 184). G6PD variants were analyzed for G6PD deficient infants only (n = 32)

**Variables**	**G6PD normal**	**G6PD deficient**	**G6PD Variants**			**P-value**
			***c.563C > T***	***c.131C > G***	**Unknown**	
n	184	32	21	3	8	
Weight (kg)	2.7 ± 0.7	2.9 ± 0.6	2.9 ± 0.5	2.6 ± 0.8	2.8 ± 1.0	0.157
Age at presentation (days)	2.2 ± 2.2	1.6 ± 1.3*	1.6 ± 1.4	1.6 ± 0.6	1.5 ± 1.1	0.215
Icterus in first 24 h of life n (%)	63(33.7)	15(46.9)	10(47.6)	1(33)	4(50)	<0.001**
Hemoglobin (g/dl)	16.5 ± 2.5	15.5 ± 1.9*	15.7 ± 2.09	13.9 ± 0.32	15.7 ± 1.8	0.172
Retic count (%)	4.0 ± 2.2	5.0 ± 2.5*	4.9 ± 2.5	5.1 ± 0.8	5.0 ± 3.2	0.140
G6PD activity (U/gHb)	14.0 ± 4.5	0.6 ± 0.9*	0.3 ± 0.2	2.2 ± 2.2	1.6 ± 2.3	<0.001**
TSH (μIU/ml)	5.9 ± 4.7	6.9 ± 4.5	7.0 ± 4.3	12.2 ± 9.2	5.1 ± 3.2	0.355
Peak bilirubin (mg/dl)	13.8 ± 4.6	16.7 ± 6.0*	16.8 ± 5.4	14.9 ± 1.2	17.2 ± 8.8	0.008**
Age at peak bilirubin (days)	4.3 ± 2.3	2.6 ± 1.9*	2.9 ± 1.6	1.6 ± 1.5	2.6 ± 2.7	0.007**
Frequency of Phototherapy (n/%)	154/82	29/90	18/86	3/100	8/100	0.701
Frequency of blood exchange (n/%)	28/15	4/12	3/14	0/0	1/13	0.934
Hospital stay (days)	6.2 ± 4.1	5.6 ± 3.1	5.2 ± 3.1	4.6 ± 1.5	7.1 ± 3.5	0.254

All values are expressed as mean ± 1SD.

*p < 0.05 between G6PD normal and deficient neonates** *P-value* < 0.05 between G6PD *c.563C > T* variant group and G6PD normal infants.

### Molecular analysis and G6PD assay

Thirty two G6PD deficient infants were tested for G6PD variants. *G6PD c.563C > T* and *c.131C > G* variants were detected in 21 (65%) and three (9%) infants respectively; the genotype of eight (25%) infants remained uncharacterized. The later include three samples that could not be fully analyzed because of insufficient DNA and five samples where the genetic variants were not ascertained at the completion of our full workup. Associated *c*.*1311 T* polymorphism was seen in four samples (19%) with *c.563C > T* variant. Infants with *G6PD c.563C > T* mutation showed markedly low levels of G6PD enzyme activity (mean ± 1SD; 0.3 ± 0.2 U/gHb) in comparison with G6PD normal infants (mean ± 1SD; 14.0 ± 4.5, p < 0.001). *G6PD* genotype of G6PD normal infants was not determined.

### Clinical and laboratory evaluation and outcome

**Figure 1:**
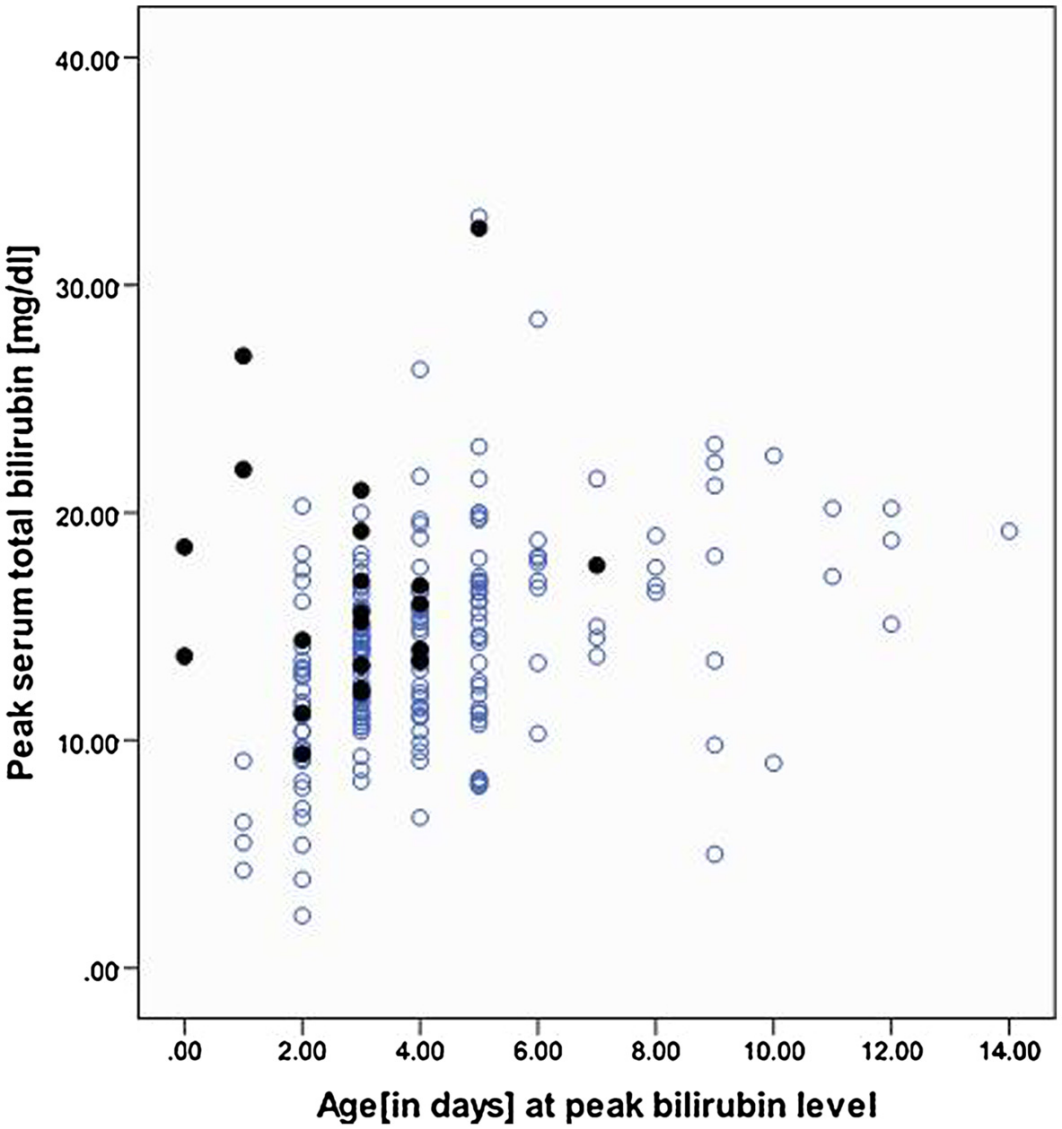
**Time to peak bilirubin in *G6PD c.563C > T *(closed circles) and G6PD normal (open circles) infants.**

**Figure 2:**
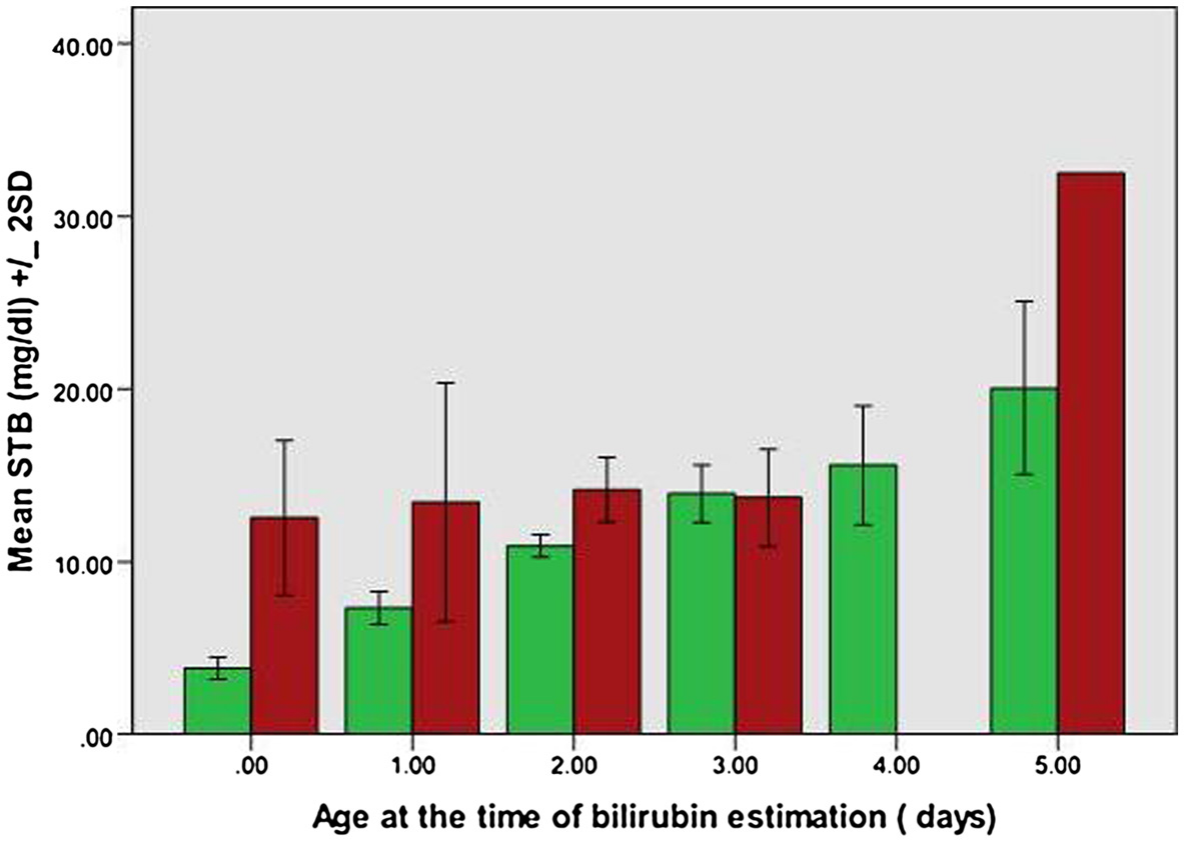
**Initial serum total bilirubin (STB) in G6PD normal (green bars) and *G6PD c.563C > T *group (red bar) as estimated during first five days of life.**

G6PD normal (n = 184) and deficient groups (n = 32) showed statistically significant differences for: age at manifestation of jaundice, peak bilirubin level and age at peak bilirubin, hemoglobin and reticulocyte count (Table 1). In contrast to G6PD normal infants, G6PD deficient infants depicted clinical jaundice earlier (mean ± 1SD; 1.6 ± 1.3 days vs. 2.2 ± 2.1 days, *p =* 0.026), reached higher peak bilirubin levels (mean ±1SD; 16.7 ± 6.0 mg/dl vs. 13.8 ± 4.6 mg/dl, *p =* 0.002) at an earlier age (mean ± 1SD; 2.6 ± 1.9 days vs. 4.3 ± 2.3 days, *p* < 0.001). The results showed that G6PD deficient neonates presented earlier consistent with the onset of overt clinical jaundice as the trigger for hospital admission. G6PD normal infants took a mean of 2.2 ± 2.2 days to develop peak jaundice from its onset in contrast to 1.6 ± 1.3 days taken by G6PD deficient infants (p-value 0.026). Hemoglobin (mean ± 1SD; 16.5 ± 2.5 vs. 15.5 ± 1.9 g/dl, p-value 0.036) and reticulocyte count (mean ± 1SD; 4.0 ± 2.2 vs. 5.0 ± 2.5, p-value 0.047) showed significant differences in the two groups. However, no statistically significant difference was seen in mean bodyweight, TSH levels, length of hospital stay and frequency of need to initiate phototherapy or blood exchange. Comparison of *G6PD c.563 > T* group (n = 21) with G6PD normal infants (n = 184) showed that these G6PD variant infants were at higher risk for early and moderate hyperbilirubinemia (Table 1). Exchange transfusion was needed in 15% of G6PD normal and 12% in G6PD deficient infants. Figure 1 showed that infants carrying *c.563C > T* mutation achieved peak bilirubin within five days of their life [except one baby] in contrast to normal infants who demonstrated peak levels dispersed between days 1–17 of their life. It was observed (Figure 2) that the initial estimated serum total bilirubin levels were significantly higher for infants inheriting *G6PD c.563C > T* when compared to G6PD normal infants during their first 72 h of life [0–3 days] of life (p-value 0.010,< 0.001 and 0.002 respectively).

There were 96 G6PD normal (57%) and nine *G6PD* c.563C > T infants (56%) who were anemic for age and additionally 33 (37%) and 3 (33%) of them respectively showed reticulocytosis. Although there were statistically significant differences in hemoglobin, haematocrit and reticulocyte count of two groups; the reported values largely represent a normal range during this immediate postnatal time frame.

The possible etiologic risk factors for hyperbilirubinemia in the G6PD normal icteric infants are listed in Table 2. Collectively, prematurity (gestational period 35 – 36 weeks) with associated sepsis constituted the most frequent pathological reason for icterus, accounting for 21% of all the causes. Mean [±SD] G6PD enzyme activity in this group (n = 39) was 13.8 ± 3.8 U/gHb but *G6PD* genotype was not determined in any infant. The etiology remained unresolved in a significant number of infants (n = 77 or 42%). These infants had normal G6PD enzyme levels [mean 14.4 ± 6.1U/gHb] but as stated before none of them was tested for *G6PD variant*. Additional risk factors for hyperbilirubinemia were observed in eight or 23% G6PD deficient infants; including pre-term (n = 4), ABO or Rh incompatibility (n = 3), and preterm with sepsis (n = 1). However, no statistically significant difference was observed when the two G6PD deficient groups [with and without confounders] were compared for age at peak bilirubin and peak bilirubin achieved [p = 0.590 and p = 0.873]. Similar confounders were present in three neonates with *G6PD c.563C > T* variant (pre-term n = 1, preterm and sepsis n = 1 and ABO incompatibility n = 1). However, statistical significance in peak bilirubin levels and age at peak bilirubin levels between G6PD *c.563C > T and G6PD normal infant was* maintained after exclusion of these three neonates.

**Table 2:** Possible etiology of hyperbilirubinemia in 184 G6PD normal male neonates

**Causes**	**Possible etiology**	**n (%)**
Isolated	Sepsis	32(17.3)
	Gestation period 35–36 weeks	26(14.1)
	ABO or Rh compatibility	8(4.3)
	Cephalhematoma	2(1.0)
	Inborn error of metabolism	1(0.5)
More than one cause	Gestation period 35–36 weeks and sepsis	39(21.1)
	Gestation period 35–36 weeks, sepsis, ABO incompatibility	1(0.5)
	Cephalhematoma and sepsis	1(0.5)
Unknown		77(41.8)
Total		184(100)

Complete recovery was observed in all infants (n = 216).

## Discussion

We characterized *G6PD* genes in 32 neonates and identified *G6PD c.563C > T* as the most frequent variant while *c.131 C > G* was less common. We studied a small population of G6PD deficiency infants but nevertheless *G6PD c.563C > T* was observed as an important risk factor for early development and moderately severe hyperbilirubinemia in neonates. Moreover, *G6PD c.563C > T* variant was also implicated in driving earlier and higher peak bilirubin levels compared to G6PD normal newborns. Additionally, at the time of recognition of hyperbilirubinemia, the serum total bilirubin was significantly higher for this variant in contrast to G6PD normal infants of the same age group.

*G6PD Mediterrenean* or *G6PD c.563C > T variant* (188 Ser → Phe) is a type II mutation and is situated in exon 6 of *G6PD gene*. Its high occurrence in neonates is no surprise as it has been previously reported from Pakistani population [21,27]. Its association with very low G6PD enzyme activity is well known and our study confirmed that. A literature review indicated a paucity of data regarding association of *G6PD c.563C > T* with the clinical course of neonatal hyperbilirubinemia. Ainoon and his colleagues studied 17 male infants with neonatal hyperbilirubinemia secondary to *G6PD c.563C > T variant*. They observed icterus in 8 infants within first 48 h of their life, with a mean peak bilirubin levels [231.8 umol/l or 13.4 mg/dl] at mean age of 3.8 days; 14% of these neonates required photo therapy [44]. However, they did not have a comparable control group of G6PD normal infants. Few individual reports described the prevalence of *G6PD c.563C > T variant* in neonates. For example, it was seen in 3 of 70 (4%) icteric Egyptian infants [12], 12 of 65 (18%) G6PD deficient Saudi infants [14] and 39 of 43 (91%) G6PD deficient Sardinian neonates [13]. All these reports lack clinical description. A few reports from Chinese population illustrated G6PD variants in neonatal hyperbilirubinemia but those reports did not demonstrate *G6PD c.563C > T* and therefore their results were not comparable to our work [11,45]. Kaplan in 2001 studied 52 infants with *G6PD c.563C > T* and observed hyperbilirubinemia in 16 (30.8%) neonates, in contrast to 10 (6%) controls [3]. The first STB value (done at 3 h) in 13 of 28 neonates was greater than or equal to mean and such infants were more likely to develop hyperbilirubinemia compared to 3 of 24 with a first STB less than the mean (relative risk 3.7). There was statistically significant difference in the rise of mean bilirubin level between cases and controls (0.15 mg/dl/h. vs. 0.13 mg/dl/h., p = 0.01).

Our study indicated statistically significant differences in hemoglobin and reticulocyte counts in G6PD deficient compared to normal infants, the reported values for both groups largely remained within the normal range for age-matched newborns. This finding is consistent with published literature related to G6PD deficiency and neonatal hyperbilirubinemia, where anemia and reticulocytosis are typically not evident [5].

*G6PD c.131 C > G* or *Orissa variant* (44 Ala → Gly) is a type III G6PD variant with mutation localized in exon 3 of G6PD gene. There is a single report of *G6PD Orissa* in one Malaysian neonate which was unassociated with icterus [44]. In contrast, three icteric neonates presented with G6PD Orissa in our study and each required phototherapy. This was unexpected as besides prematurity in one infant, no associated confounder was identified.

Several risk factors were identified in G6PD normal infants. The main risk factor for jaundice was identified as prematurity either with or without sepsis in G6PD normal infants. This finding corroborates previously published reports describing a high risk of bilirubin production-conjugation imbalance in borderline premature infants [46]. Sepsis which is a leading cause of neonatal mortality in developing countries [47] carries a high risk for jaundice in preterm infants in contrast to term infants [48]. Etiology of jaundice was unidentified in 42% of our G6PD normal infants and the possibility of G6PD variants could not be entirely excluded, as analysis for subtle G6PD variants was not made in G6PD normal infants. Confounders were observed in 23% G6PD deficient infants, including sepsis, prematurity and Rh incompatibility. However, there was no statistical difference in the peak bilirubin levels and age at peak bilirubin level in the G6PD deficient groups with or without confounders (p = 0.873 and 0.590 respectively). This suggests a lack of significant contribution of confounders towards hyperbilirubinemia in G6PD deficient infants. The absence of significant difference in the frequency of exchange transfusion in G6PD normal and deficient infants might be attributed to small sample size of G6PD deficient group. In addition, G6PD normal infants requiring exchange transfusion (n = 28) had comorbid like septicemia and prematurity (25/28 or 89%) which would have contributed towards significant hyperbilirubinemia and hence a need for exchange transfusion.

### Strengths and limitations

This study underscores the correlation of *G6PD c.563C > T* with neonatal hyperbilirubinemia. Few limitations were the small sample size of infants with G6PD deficiency and absence of G6PD variants analysis in G6PD normal infants. This approach likely underestimated the contribution of G6PD deficiency in the genesis of hyperbilirubinemia as G6PD deficiency could certainly be co-expressed in the background of other triggers, such as mild prematurity or sepsis. The study was done in hospitalized infants in whom timely initiation of appropriate management was performed. In Pakistan, 65% of births occur at home and a selection bias may be at play obscuring the true picture of the extent of problem.

## Conclusions

We concluded that *G6PD c.563C > T* mutation carried a risk of early development of moderate hyperbilirubinemia in neonates. A large prospective and community based study is required to elucidate the true burden of *G6PD c.563C > T* and its association with neonatal icterus.

## Competing interests

Authors declare that they have no competing interests.

## Authors’ contributions

BM conceived of the study, participated in its design and coordination and wrote manuscript. AN performed the mutational analysis, studied the sequence alignment, and contributed to manuscript preparation. SAK1 participated in study design, collection and analysis of the data. SAK2 collected and analyzed the data and participated in study design. MQ participated in study design, conception and drafting of manuscript. All authors read and approved the final manuscript.

## Pre-publication history

The pre-publication history for this paper can be accessed here:

http://www.biomedcentral.com/1471-2431/12/126/prepub
