# Erratum to: Neonatal Hyperbilirubinemia in infants with *G6PD c.563C > T Variant*

**DOI:** 10.1186/s12887-016-0752-1

**Published:** 2016-12-09

**Authors:** Bushra Moiz, Amna Nasir, Sarosh Ahmed Khan, Saleema Amin Kherani, Maqbool Qadir

**Affiliations:** 1 Department of Pathology and Microbiology, The Aga Khan University Hospital, Karachi, Pakistan; 2 Medical Student, The Aga Khan Medical College, Karachi, Pakistan; 3 Department of Pediatrics and Child Health, The Aga Khan University, Karachi, Pakistan

## Erratum

Since the publication of this article [1], author Salima Kherani has changed the spelling of her name to Saleema Kherani. The correct author’s name has been included to the authors’ list of this erratum.
